# Quality versus quantity: Foraging decisions in the honeybee (*Apis mellifera scutellata*) feeding on wildflower nectar and fruit juice

**DOI:** 10.1002/ece3.2478

**Published:** 2016-09-20

**Authors:** Kyle Shackleton, Nicholas J. Balfour, Hasan Al Toufailia, Roberto Gaioski, Marcela de Matos Barbosa, Carina A. de S. Silva, José M. S. Bento, Denise A. Alves, Francis L. W. Ratnieks

**Affiliations:** ^1^ Laboratory of Apiculture and Social Insects School of Life Sciences University of Sussex Brighton UK; ^2^ Departamento de Entomologia e Acarologia Escola Superior de Agricultura “Luiz de Queiroz” Universidade de São Paulo Piracicaba São Paulo Brazil; ^3^ Departamento de Biologia Faculdade de Filosofia Ciências e Letras de Ribeirão Preto Universidade de São Paulo Ribeirão Preto São Paulo Brazil

**Keywords:** central place forager, crop sugar, forage quality, foraging behavior, nectar concentration, *Psidium guajava*, sugar–water balance

## Abstract

Foraging animals must often decide among resources which vary in quality and quantity. Nectar is a resource that exists along a continuum of quality in terms of sugar concentration and is the primary energy source for bees. Alternative sugar sources exist, including fruit juice, which generally has lower energetic value than nectar. We observed many honeybees (*Apis mellifera scutellata*) foraging on juice from fallen guava (*Psidium guajava*) fruit near others foraging on nectar. To investigate whether fruit and nectar offered contrasting benefits of quality and quantity, we compared honeybee foraging performance on *P. guajava* fruit versus two wildflowers growing within 50 m, *Richardia brasiliensis* and *Tridax procumbens*. Bees gained weight significantly faster on fruit, 2.72 mg/min, than on either flower (0.17 and 0.12 mg/min, respectively). However, the crop sugar concentration of fruit foragers was significantly lower than for either flower (12.4% vs. 37.0% and 22.7%, respectively). Fruit foragers also spent the most time handling and the least time flying, suggesting that fruit juice was energetically inexpensive to collect. We interpret honeybee foraging decisions in the context of existing foraging models and consider how nest‐patch distance may be a key factor for central place foragers choosing between resources of contrasting quality and quantity. We also discuss how dilute solutions, such as fruit juice, can help maintain colony sugar–water balance. These results show the benefits of feeding on resources with contrasting quality and quantity and that even low‐quality resources have value.

## 1 Introduction

Foraging decisions are often based on increasing the rate or efficiency of food collection (Cowlishaw, 1997; Houston & McNamara, 2014; Scrimgeour & Culp, 1994; Waite & Ydenberg, 1994; Wang, Ings, Proulx, & Chittka, 2013). In particular, increasing the quality and quantity of food consumed, or consuming an optimal amount (Bunning et al., 2015), will increase fitness. However, food patches in the environment can vary greatly (Dennis, 2010; Pickett & Cadenasso, 1995), such that animals are faced with decisions between resources of varying quality and quantity. Quality–quantity trade‐offs drive the seasonal movements of some animals (Bischof et al., 2012; Van Beest, Mysterud, Loe, & Milner, 2010; Van der Wal et al., 2000). However, central place foragers are more restricted in their foraging movements and must base their foraging decisions on what is available nearby.

Nectar is an ideal resource for studies of food quality and quantity in central place foragers for several reasons. First, nectar is consumed by many central place foragers, such as bees, which can be easily observed in the field and individually marked. Second, nectar is composed predominantly of sugar and water, allowing quality to be quantified in energy units (Heil, 2011). Nectar also contains other nutrients such as amino acids, but these are normally present in small amounts which are of secondary importance for bees assessing flower quality (Hendriksma, Oxman, & Shafir, 2014). Third, both volume and sugar content of nectar vary greatly among species and with environmental conditions, giving bees many choices (Corbet, Unwin, & Prŷs‐Jones, 1979; Heil, 2011). Southwick, Loper, and Sadwick (1981) and Seeley (1986, 1995) found similar nectar concentrations in surveys of flowers and bee crops, respectively, ranging from 15% to 65% and 18% to 68% sugar concentration, respectively. There are also numerous alternative sugar sources on which bees occasionally feed including honeydew, honey robbed from other bee colonies, and fruit juice (Santas, 1983; Wäckers, 2005; Winston, 1987).

Fruit is an important food resource for many birds and mammals, which may also act as seed dispersers (Howe, 1986). Indeed, fruit often serves a similar function to nectar in that it offers a reward to animal mutualists in plant reproduction. Many species of insect, including social insects, also forage on fruit (Evison & Ratnieks, 2007; Helanterä & Ratnieks, 2008; Jander, 1998; Noll, Zucchi, Jorge, & Mateus, 1996). In São Paulo State, Brazil, we have observed honeybees foraging on fallen *Mangifera* spp. (mango) and *Psidium guajava* (guava) fruit juice within meters of those foraging for nectar on flowers. However, in comparison with nectar, the sugar concentration of fruit juice appears to be low. White and Stiles (1985) analyzed the sugar content of 37 fruit species, 27 of which fell within the range of 10%–25%, far lower than most nectars.

Honeybees can fly considerable distances to food sources (Beekman & Ratnieks, 2000; Couvillon, Schürch, & Ratnieks, 2014; Ratnieks & Shackleton, 2015), and colonies show great ability to direct their forager workforce to the most rewarding sources (Seeley, Camazine, & Sneyd, 1991). Why, therefore, do they gather dilute fruit juice when nectar is available? One possibility is that fruit is near the end point of quality versus quantity continuum. Individual flowers contain only small nectar volumes, meaning a bee may need to visit hundreds of flowers to fill its crop (Balfour, Gandy, & Ratnieks, 2015). By contrast, a single fruit could contain enough juice to fill the crop many times over, but at a lower sugar concentration. This leads to the prediction that fruit juice can be collected more efficiently than nectar, thereby increasing energetic reward. Additionally, the water content of fruit juice may itself have value if water demand is high as it is used in thermoregulation and the dilution of honey before feeding.

To test whether resources had contrasting benefits of quality and quantity, we compared the foraging performance of honeybees on fallen *P. guajava* fruits versus two species of wild flowers growing nearby. First, we measured the rate of weight change of bees foraging on each resource to test the prediction that fruit foragers would gain weight faster than flower foragers. Second, we measured the energetic quality of each resource in terms of its sugar content, to confirm that guava juice was more dilute than the nectar alternatives. Third, we quantified the activity patterns of bees on each resource, to test the prediction that fruit foragers spend more time handling and less time on the energetically expensive activity of flying between food items.

## 2 Methods

### 2.1 Study site and species

The study was carried out on the campus of the Luiz de Queiroz College of Agriculture (Escola Superior de Agricultura Luiz de Queiroz, ESALQ), Piracicaba, São Paulo State, Brazil, between 22 February and 6 March 2015. The weather conditions were good for honeybee foraging throughout the day. There was little cloud, rain, or wind, and daytime temperatures ranged from 21.8 to 36.2°C (mean ± *SD* 26.5 ± 2.9°C. All temperature data were gathered from a weather station on the campus (22°42′30′′W, 47°38′00′′S, altitude 546 m) approximately 850 m from our study area.

**Figure 1:**
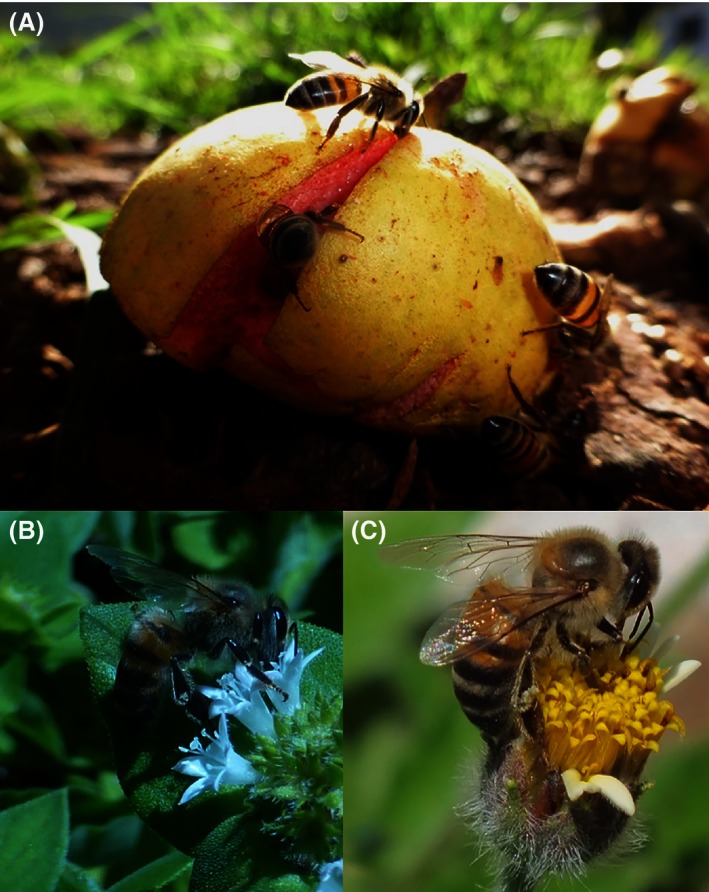
Honeybees (*Apis mellifera scutellata*) foraging on a (A) fallen *Psidium guajava* fruit, (B) *Richardia brasiliensis,* and **(C)** *Tridax procumbens* on the ESALQ campus, São Paulo State, Brazil, March 2015

We studied worker honeybees, *Apis mellifera scutellata*, foraging on three sugar resources: fruit fallen from guava trees, *Psidium guajava* (Myrtaceae), and two species of wild flowers, *Tridax procumbens* (Asteraceae) and *Richardia brasiliensis* (Rubiaceae), growing in patches in lawns (Figure 1). These two species were chosen as they were abundant, in full‐bloom, growing near (<50 m), the guava trees, and were attracting many honeybees as well as other insects.

### 2.2 Rate of forager weight gain

We determined the rate at which honeybees collected nectar or fruit juice by measuring weight gains of individual bees foraging exclusively on each of the three resources. We first marked bees foraging on each resource with paint dots or numbered tags (Opalithplättchen, manufactured by Chr Graze, Endersbach, Germany) for individual identification. We did not select bees carrying pollen in their baskets, and therefore, only sampled nectar or juice foragers. From a few hours to a few days later, we would capture a previously marked bee at one of the foraging sites in a plastic tube. We captured bees as they were seen and did not know how long they had previously spent foraging. We then weighed the bee plus tube to ±0.1 mg using an Ohaus Explorer balance. Within a few minutes, the bee was released back at her foraging patch and allowed to resume foraging for 5–10 min. During this period, the bee was constantly observed to ensure that she was foraging actively and exclusively on that resource. She was then recaptured and reweighed to determine her weight change per unit time. The procedure caused almost half of bees (49%) to become agitated and they left the patch immediately when released after the first weighing. A further 4% initially continued to forage but left the patch before 5 min, indicating that they had completed their foraging bout. No data were taken from either of these groups. The remaining bees immediately resumed foraging. In total, we obtained data from 22, 25, and 22 bees on *P. guajava*,* R. brasiliensis,* and *T. procumbens,* respectively.

### 2.3 Forage quality

Nectar in both flower species was present in too small a volume to be extracted with a microcapillary tube. Instead, we measured the sugar concentration in the honey stomachs (crops) of bees foraging exclusively on each resource (*n* = 20 per resource). A bee was captured in a plastic tube and then chilled for 10 min at 4°C to cause it to stop moving. We squeezed the abdomen gently with fingers to cause the regurgitation of a small drop of juice or nectar and used a refractometer, Bellingham–Stanley “Eclipse” 0–50% sugar, to determine sugar concentration.

### 2.4 Time and activity budgets of foragers

We quantified the activity patterns of foragers (*n* = 20 per resource) by following individual bees with a stopwatch and digital camcorder (Sony HDR‐CX115) for one minute. We recorded how many food items, flowers, or fruits were visited and the proportion of time spent handling versus walking or flying between food items. Handling was defined as a bee orientating itself on a food item and probing with its proboscis.

### 2.5 Effect of temperature

Ambient temperature can affect the foraging behavior of bees. To investigate any influence of temperature on forager weight gain, crop sugar concentration, visitation rate, or time spent handling, we obtained data from the weather station (see Study site and species). This recorded temperature at 15‐min intervals throughout the day.

### 2.6 Resource quality in the wider environment

To gauge overall quality of resources being collected by honeybees, we studied workers returning to three hives in an apiary within 100 m of all resource patches. To determine the average weight at the start of a foraging trip, we collected and individually weighed 60 exiting bees from each hive. We also collected 60 returning bees per hive, 30 at 10:00 (temperature 26.4 ± 0.5°C) and 30 at 15:30 (31.6 ± 0.9°C) on three consecutive days. Each bee was individually weighed, checked for pollen in its baskets, and had the sugar concentration of the liquid in its crop determined, as above.

### 2.7 Daytime pattern of resource use

To compare the temporal use of fruit versus flowers, we counted honeybees on the three resources every 30 min for 3 days during daylight hours, 06:30 to 19:00. We surveyed five nearby patches of each resource. *P. guajava* patches contained fruit in various stages of decay, but each had some recently fallen fruit which were attracting insects. Flowers were in full‐bloom and growing in distinct patches that formed a monoculture or near monoculture. Each patch was approximately equal in size, 1.0 m^2^, but as they were natural patches they were not precisely equal. This was not a problem as our aim was not to quantify the density of insects on the resources but their temporal use. A rapid count of the bees present on each patch was made, so that the count reflected the number currently on the patch (Garbuzov & Ratnieks, 2014). Only bees visiting flowers or fruit were counted.

### 2.8 Statistical analysis

We tested for the effect of foraging resource (*P. guajava*,* R. brasiliensis,* and *T. procumbens*) on four measures of honeybee foraging performance: rate of weight change, crop sugar concentration, visitation rate and proportion of time spent handling. Following transformation, the data did not meet the parametric assumptions. Therefore, we used the nonparametric Kruskal–Wallis ANOVA. We made *post hoc* pairwise comparisons between resources using Mann–Whitney–Wilcoxon test and applied a Bonferroni correction. To investigate any influence of temperature, we performed Spearman's rank‐order correlations between each of our four variables and temperature. We also investigated the relationship between the weight of foragers returning to their hives and their crop sugar concentration. Again the data did not meet the parametric assumptions. Therefore, we used Spearman's rank‐order correlation. All analyses were performed using R version 3.1.1 (R Core Team 2014).

## 3 Results

### 3.1 Rate of forager weight gain

**Figure 2:**
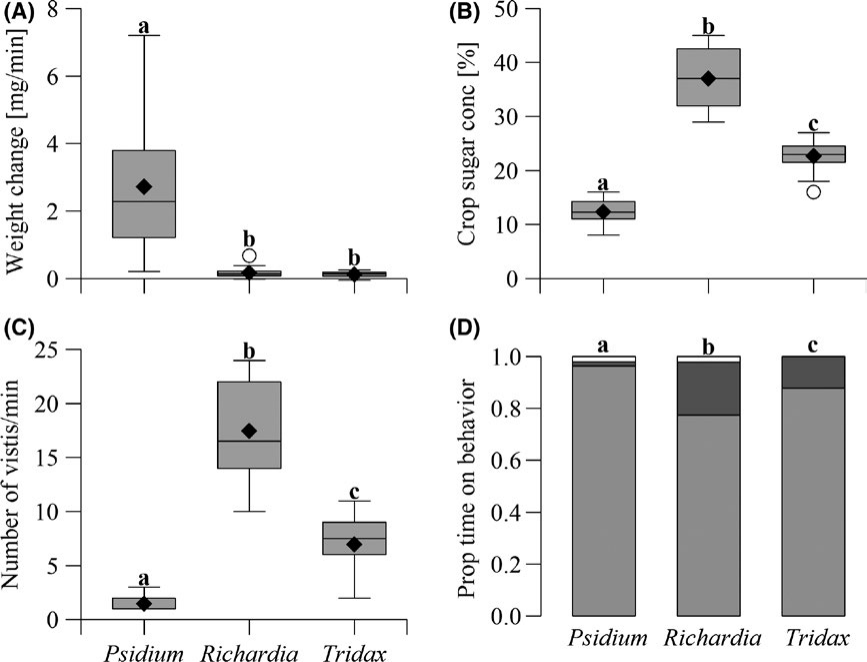
Honeybee foraging performance on the three different resources. (A) Honeybees gained weight at a faster rate while foraging for juice on *Psidium guajava* fruit than on nectar from either *Richardia brasiliensis* or *Tridax procumbens* flowers. (B) Crop sugar concentration was significantly higher in honeybees foraging on both flowers than on fruit and highest in *R. brasiliensis*. (C) Number of food items, flowers, or fruit, visited during one minute, was significantly lower for honeybees foraging on fruit than either flower species. (D) Bees on fruit spent a greater proportion of their time handling than on either flower, light gray = handling, dark gray = flying, white = walking, flying, and walking not analyzed. Whiskers indicate 1.5× the interquartile range, circles indicate outliers, diamonds indicate means, and letters indicate significant differences based on *post hoc* Mann–Whitney‐Wilcoxon test with Bonferroni correction

Honeybees foraging on *P. guajava* fruit gained weight at 2.72 ± 1.86 mg/min (mean ± *SD*), *n* = 22, significantly greater by factors of 16 and 22 times than those foraging on *R. brasiliensis* or *T. procumbens* flowers, respectively (Table 1, Figure 2A). One bee actually lost weight while foraging on *R. brasiliensis*, indicating that weight gains on this resource were marginal. There was no significant difference between the two flowers. There was no effect of temperature (Spearman's rank‐order correlation, *p* = .36, *rho* = 0.11).

**Table 1:** Test statistics for honeybee weight change, crop sugar concentration, visitation rate, and proportion of time spent handling while foraging on three resources

Response variable	Kruskal–Wallis ANOVA			MWW pairwise comparison *p* values		
	*p*	*Χ* ^2^	*df*	*Psidium*:*Richardia*	*Psidium:Tridax*	*Richardia:Tridax*
Weight change	<.001	42.76	2	<.001	<.001	.6314
Crop sugar	<.001	52.47	2	<.001	<.001	<.001
Visitation rate	<.001	77.42	2	<.001	<.001	<.001
Proportion of time handling	<.001	35.82	2	<.001	<.001	<.001

Kruskal–Wallis ANOVAs show differences among groups. Mann–Whitney‐Wilcoxon (MWW) tests compare specific pairs within groups.

### 3.2 Forage quality

The mean sugar concentration in the crops of worker honeybees foraging on *P. guajava* was 12.4 ± 2.2%, *n* = 20, similar to other fruits (White & Stiles, 1985). This was significantly lower than the nectar concentrations taken from the crops of honeybees foraging on *R. brasiliensis*, 37.0 ± 5.4%, *n* = 20, or *T. procumbens* 22.7 ± 2.9%, *n* = 20 (Table 1, Figure 2B). All pairwise comparisons were significant. There was no effect of temperature (Spearman's rank‐order correlation, *p* = .22, *rho* = −0.16).

### 3.3 Time and activity budgets of foragers

Foraging bees visited significantly fewer *P. guajava* fruits per minute, 1.47 ± 0.63, *n* = 20, than either *R. brasiliensis* or *T. procumbens* flowers, 17.47 ± 4.13, *n* = 20, and 6.97 ± 2.67, *n* = 20, respectively (Table 1, Figure 2C). This was not due to long search times for fruit. Bees on *P. guajava* spent a significantly greater proportion of their time handling than bees on either flower (Table 1, Figure 2D), meaning less time moving between food items (Figure 2D, data not analyzed statistically). Most fruit foragers (60%) remained feeding with their proboscis extended on a single fruit for the entire duration of the one minute foraging observation period. All pairwise comparisons were significant. The mean wet mass of fallen fruits was 28.9 ± 15.2 g, *n* = 10, so that a single fruit would contain many times the juice needed to fill a honeybee crop with c. 30 μl liquid. By comparison, nectar volumes of both flowers were so small that we could not retrieve any using microcapillary tubes for analysis. There was no effect of temperature on visitation rate (Spearman's rank‐order correlation, *p* = .99, *rho* = −0.00) or proportion of time handling (*p* = .66, *rho* = −0.06).

### 3.4 Resource quality in the wider environment

Bees departing hives had a mean weight of 59.1 ± 4.3 mg, *n* = 60. Of the 180 bees captured returning to their hives, 40, 22%, had empty crops. In seven of these, pollen was present in the pollen baskets, indicating they had been foraging for pollen alone. The other 33, 18%, were presumably unsuccessful foragers or had left the hive for other purposes such as defecation, orientation, or undertaking. There was no difference in weight gain between bees collected in the morning and afternoon (Mann–Whitney–Wilcoxon test, *p* = .45).

**Figure 3:**
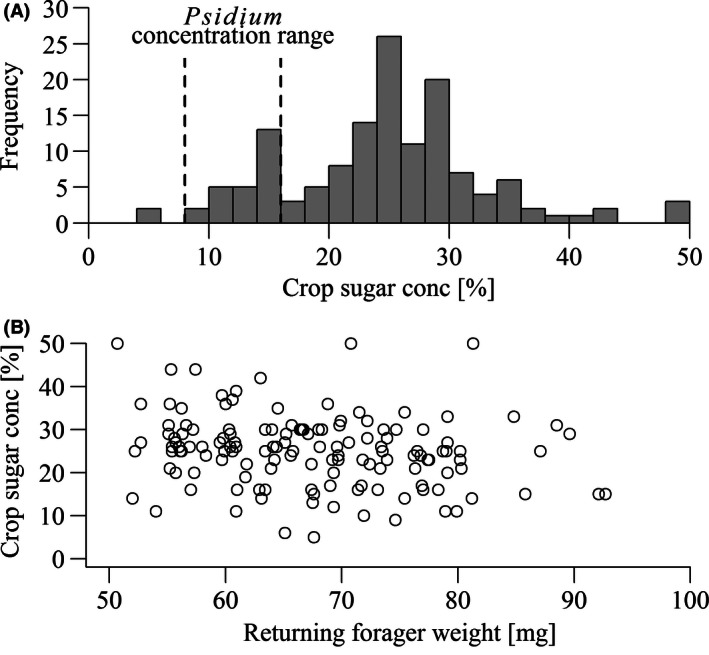
(A) Distribution of crop sugar concentrations of 140 honeybee foragers with liquid in their crops returning to hives near (<100 m) the resource patches. Vertical dashed lines indicate the range of sugar concentrations found in crops of honeybees foraging on *P. guajava* (Figure 2B). (B) Correlation between returning forager mass and the concentration of sugar solution present in their crops. Heavier foragers had lower crop sugar concentrations

In the 140, 78%, of the returning bees with liquid in their crop, sugar concentrations ranged from 5% to 50% (the limit of our refractometer was 50%, but only three bees were ≥50%) with a mean of 25.2 ± 8.3%, *n* = 140 (Figure 3A). Of these bees, 18% had crop sugar concentrations within the range for honeybees foraging on *P. guajava* fruit (8%–16%, Figure 2B). This was outside the range of concentrations found in bees foraging on either flower except for one *T. procumbens* outlier. Figure 3A shows a small peak within this range. Only two bees had concentrations <8%. The mean weight of the 78% bees with crop sugar was 67.4 ± 9.3 mg, *n* = 140, an 8.3 mg, 14%, gain over departing foragers. There was no difference in crop sugar concentration between bees collected in the morning and afternoon (Mann–Whitney–Wilcoxon test, *p* = .22).

Returning forager weight had a weak, negative, but significant correlation with crop sugar concentration (Spearman's rank‐order correlation coefficient = −0.22, *p* = .009, *n* = 140, Figure 3B), indicating that bees foraging on higher quality resources were more likely to return with a lower weight of resource.

### 3.5 Daytime pattern of resource use

**Figure 4:**
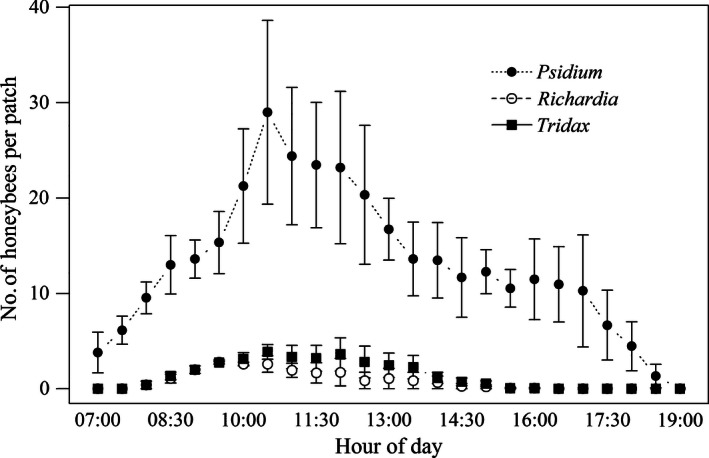
Mean number of honeybees foraging on five 1‐m^2^ patches each of *P. guajava*,* R. brasiliensis,* and *T. procumbens* throughout three good foraging days from 3 to 5 March 2015. Sunrise 06:03–06:04, sunset 18:34–18:32. Error bars ± 1 *SE*

Honeybee foraging on all three resources peaked in the morning, between 09:30 and 12:00, before declining in the afternoon (Figure 4). Bees were able to exploit fruit for the whole day and for a longer period than for either flower. A small number of bees were already foraging on fruit at 07:00 when observations began shortly after sunrise (06:03–06:04), while the first bees arrived on the flowers later at 07:30. Foraging had ceased on both flowers by 15:30 (8 h) while bees continued to forage on fruit until 18:30 (12.5 h), at sunset (18:34–18:32). Thus, fruit foraging occurred for 56% longer each day than nectar.

## 4 Discussion

Honeybees gathered fruit juice at a faster rate than nectar. However, as expected, fruit juice was of lower sugar concentration than nectar. This supports our prediction of fruit juice and nectar being at opposite ends of a quality–quantity continuum.

Weight gains of foraging honeybees were broadly comparable with those of Dukas and Visscher (1994), who calculated an average of approximately 0.7 mg/min. This was less than for fruit foragers (2.72 mg/min), but faster than our nectar foragers (0.17 and 0.12 mg/min). The rapid weight gain of bees on fruit was probably due to the large volumes of juice available in individual fruits compared with the small amounts of nectar in individual flowers. As fruit juice had lower sugar concentration than nectar, it was probably also less viscous. Lower viscosity generally leads to higher intake rates in insects (Borrell, 2006; Roubik & Buchmann, 1984) and honeybees may preferentially forage on less viscous solutions when sugar concentration is constant (Nicolson, de Veer, Köhler, & Pirk, 2013). Fruit foragers moved relatively little between fruits and, therefore, were able to spend proportionally more time handling. As handling requires less energy than flight (reviewed in Balfour et al., 2015), this suggests that foraging on fruit juice incurred lower energetic costs than on nectar.

The crop contents of foragers returning to their hives (Figure 3A) indicated a wide range in the concentration of sugar resources being collected. However, average weight gain was low (8.3 mg). Seventeen percent of bees had crop contents with sugar concentration in the range of *P. guajava*, 8%–16% sugar, a far higher proportion than found in this range by Seeley (1986). Honeybee colonies direct their foraging toward the most rewarding sites (Seeley, 1995; Seeley et al., 1991) and although we have no knowledge of the overall foraging landscape, low‐quality resources were nonetheless important to these colonies. The negative correlation between returning forager weight and crop sugar concentration also indicates that low‐quality resources were collected in higher volumes. Differences in reward among resources may have led to their abandonment at different levels of crop‐filling (Kacelnik, Houston, & Schmid‐Hempel, 1986; Nuñez, 1982; Schmid‐Hempel, Kacelnik, & Houston, 1985). Finally, fruit foraging was limited only by daylight (Figure 4), providing an important advantage over the flowers which closed or withered each afternoon.

Combining the results for weight gain and resource concentration, the rate of sugar (energy) collection on the patch should have been much higher for *P. guajava* than either *R. brasiliensis* or *T. procumbens*: Concentration of sugar solution × collection rate (Figure 1A,B) is 12.35% × 2.72 mg/min = 0.34 mg sugar min^−1^ for *P. guajava*, 37.00% × 0.17 mg/min = 0.06 mg sugar min^−1^ for *R. brasiliensis*, and 22.65% × 0.12 mg/min = 0.03 mg sugar min^−1^ for *T. procumbens*, differences greater than fivefold and 10‐fold. However, the currency animals use to measure resource profitability helps determine their foraging strategy (Houston & McNamara, 2014). Honeybees do not use gross or rate of energy gain as their criterion of profitability. Rather, they use net energetic efficiency (NEE), that is, energy gained divided by energy expended (Seeley, 1994).

**Figure 5:**
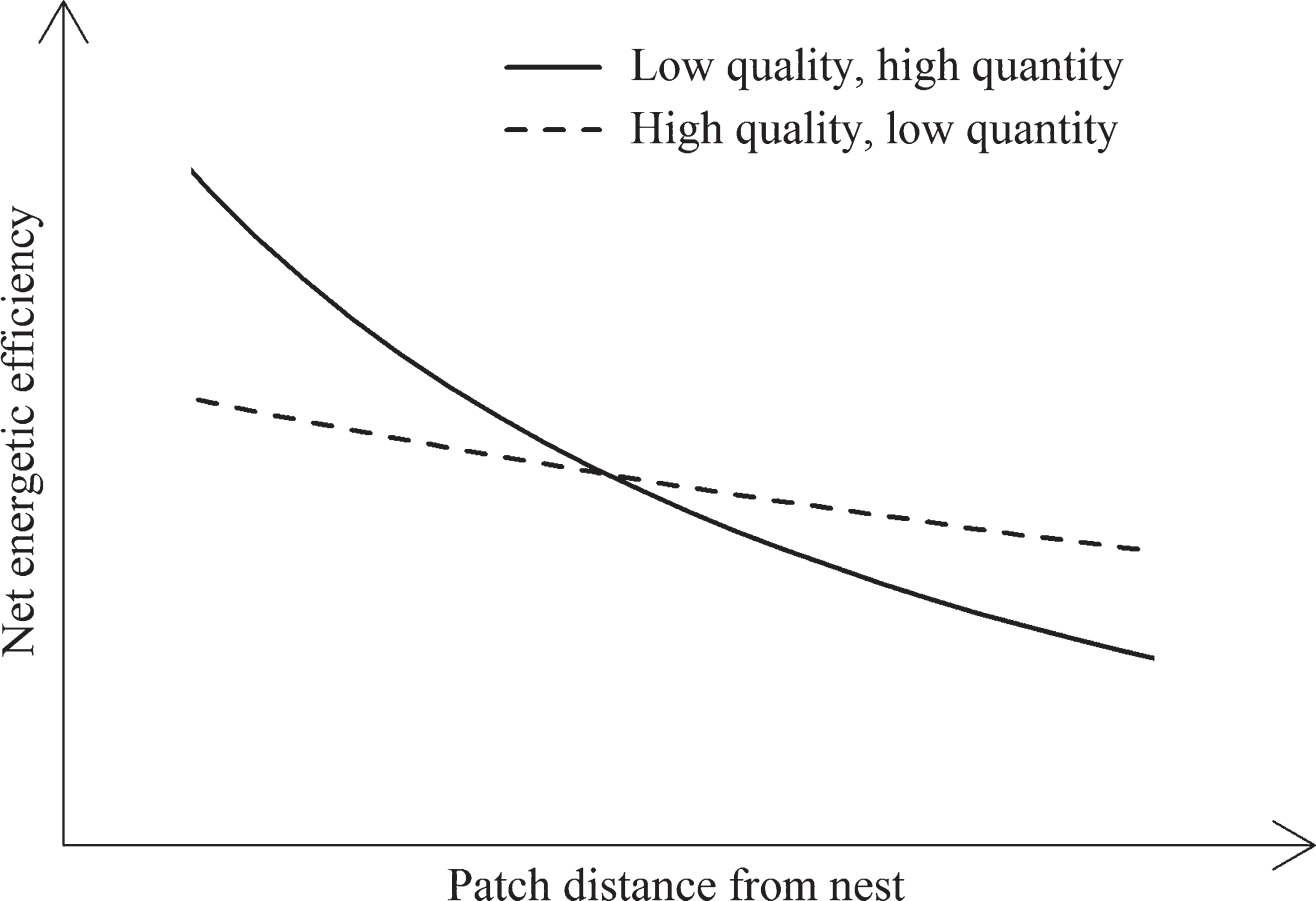
Net energetic efficiency (NEE) of a central place forager on patches of two resources differing in energy content and quantity, as a function of increasing distance from the nest. The high‐energy food provides more energy for a given volume collected, while the low‐energy and high‐quantity food have lower collection costs. When nest‐patch distance is low, the commute costs, and thus total costs, are low for the low‐energy and high‐quantity food. Its NEE is therefore high. As nest‐patch distance increases, the commute cost forms a greater proportion of the total costs. Therefore, the NEE of the low‐energy/high‐quantity food declines at a faster rate than the high‐energy and low‐quantity food. At longer distances, the NEE of the low‐energy and high‐quantity food becomes the lower, because its advantage of being quick to collect diminishes

For a given volume of solution collected, the energy gain will be greater on nectar than fruit (Figure 2B). The cost is then divided between energy spent on the patch and the commute (Pyke, 1980). We did not measure the energy bees expended, but can assume that foraging on fruit was less costly than nectar because fruit foragers would be able to fill up faster (Figure 2A) and do so using less flight, the most energetically most expensive behavior (Balfour et al., 2015; Figure 2D). This gives fruit an advantage over nectar as the energy spent on the patch would be comparatively low. However, as a central place forager, the distance from nest to patch is also a key component in determining NEE. When nest‐patch distance is low, the energy spent commuting, and thus the total cost of fruit foraging, is also low. As nest‐patch distance increases, the energy spent on the patch forms a smaller a proportion of the total cost. The NEE of fruit foraging therefore declines at a faster rate than nectar as the advantage of fruit diminishes with increasing distance (Figure 5).

This argument may be viewed in terms of a prey‐choice model (Charnov & Orians, 1973; Stephens & Krebs, 1986). In such a model, there is a trade‐off between search and handling times of prey items, and prey is handled if the energetic gain is greater or equal to the expected gain from searching for another prey item. Similarly, a bee may decide to forage on a known resource if the expected NEE exceeds its current threshold for foraging, based on social information. For a central place forager maximizing NEE, the ‘handling time’ of classical foraging models may be substituted for the energy spent on the patch and the commute. Greater nest‐patch distance leads to higher ‘handling energy’. As distance increases, the NEE of a low‐quality–high‐quantity resource diminishes more rapidly than a high‐quality–low‐quantity resource. Hence, the bee is less likely to forage on that resource as the NEE of doing so falls below the foraging threshold.

We cannot be sure where the study bees on our patches were nesting and honeybees have a large foraging range (Beekman & Ratnieks, 2000). However, there were no managed hives on the ESALQ campus (an area over 9 km^2^) other than our study hives, and most honeybees forage within 2 km of their nest (Couvillon et al., 2014). Furthermore, we inspected our study patches 1 year later when all but one hive had been removed and observed far fewer honeybees. Therefore, it seems likely that it was mainly bees from the nearby (<100 m) apiary foraging on the study the resource patches. The interaction between resource quality, quantity, and distance would merit further empirical study.

Given the high NEE of nearby fruit (Figure 5), we might expect bees to utilize it more than our returning forager data (Figure 3A) indicated. However, we compared foraging on only three resources, whereas colony foraging would likely also have detected more rewarding nectar resources in the landscape. Fruit might also involve specific advantages and disadvantages. Pollen, the primary protein source for bees, is often collected alongside nectar at flowers (Brian, 1957; Rasheed & Harder, 1997), but fruit offers no opportunity to do this. Similarly, nectar and fruit may differ in their content of amino acids and other secondary compounds and make one more rewarding than the other (Couvillon et al., 2015; Hendriksma et al., 2014). Fermentation and microbial communities present in fruit could both also incur metabolic costs and impair behavior (Bozic, Abramson, & Bedencic, 2006). Freshly fallen fruit, probably not yet fermenting, appeared the most attractive to bees. Dilute fruit juice solutions may, however, be advantageous in warm climates, as it could reduce the need for dedicated water collection (Robinson, Underwood, & Henderson, 1984).

Water plays an important role in bee colonies. The evaporation of water is used to cool both individuals and the nest as a whole and to dilute stored honey before feeding (Lindauer, 1955; Nicolson, 2009; Ostwald, Smith, & Seeley, 2016). However, there was a marked absence of dedicated water collection by colonies (Figure 3A) in comparison with the results of Seeley (1986), while one of the sugar concentration peaks of returning foragers was in the range of *P. guajava* juice. Given the hot temperatures during the study (see Study site and species), demand for water would likely be high. This leads to an additional explanation of our results. Bees may have fed on fruit because of, not despite, it being dilute. From the bees’ perspective, the most important resource shifts with environmental conditions and colony demands. The notion of a solution's quality would therefore, not necessarily be synonymous with high carbohydrate content, as the water itself has value.

The colony‐level regulation of the sugar–water balance is important in social insects. Dussutour and Simpson (2008) showed that *Rhytidoponera metallica* ants shifted their individual and colony‐level foraging efforts to match colony nutritional requirements. Following starvation, ants initially foraged more on a concentrated sugar solution, but then switched their focus to collecting more dilute solution. This enabled the ants to replenish their sugar supply and maintain a colony‐level homeostasis of sugar–water balance. These results draw parallels with our own, as a honeybee colony also regulates its forager distribution based on its nutritional requirements (Camazine, 1993). Therefore, our bees may have been displaying a compensatory feeding response akin to the ants. However, as our data are only a snapshot in time, we cannot draw a firm conclusion.

The quality–quantity and water balance hypotheses are not mutually exclusive. Fruit foraging may have offered high NEE and aided in water balance. We cannot be certain whether water was in demand, as bees may then have simply collected water, as sources were available in the landscape. These hypotheses could be teased apart experimentally by attaching water feeders to hives and measuring consumption from the feeder and the crop contents of returning foragers.

Foraging animals may consume multiple food items which vary in nutritional content in order to meet their nutritional requirements (Belovsky, 1978). In simple terms, bees forage for sugar (nectar, honeydew, fruit juice), protein (pollen), and water, although other micronutrients may also be relevant. We excluded pollen foragers and studied foragers on single resources, but both sugar and water can be viewed as separate, functionally important nutrients for the colony, even when they are collected from the same food source. In this way, a bee colony may aim for an optimum ‘intake target’ of sugar and water (Raubenheimer & Simpson, 1993; Simpson & Raubenheimer, 1993) and distribute its foragers appropriately among resources which vary in their content of these nutrients.

A low‐energy diet can reduce fitness in animals (Birkhead, Fletcher, & Pellatt, 1999; Sterner, 1993), but see Cruz‐Rivera and Hay (2000). The ability of honeybees to process dilute solutions into concentrated honey circumvents this problem, although processing honey incurs its own metabolic costs. This allows low‐energy items to be incorporated into the bee diet provided they can be collected in sufficient quantity.

Nectar dearth can lower the acceptance threshold for food, allowing honeybees to consider less rewarding or risky resources (Seeley, 1994) including robbing (Downs & Ratnieks, 2000). Similarly, nesting birds may provision their young with lower quality food when demand is high (Wright, Both, Cotton, & Bryant, 1998). Several lines of evidence suggest that there was indeed nectar dearth during the study period. Flower foragers gained weight at a low rate, foragers returning to hives gained only a small amount of weight on average (8.3 mg), and the study was conducted during late summer–early autumn, a natural period of reduced flower numbers.

Fruit foraging may well be opportunistic on the part of honeybees, occurring mainly when fruit is abundant, nearby, and during periods of either nectar dearth or high water demand (Kühnholz & Seeley, 1997). These periods need not be mutually exclusive. The ability of honeybee colonies to scour the landscape, communicate the location of the most rewarding patches, and rapidly shift their foraging (Donaldson‐Matasci, DeGrandi‐Hoffman, & Dornhaus, 2013; Seeley, 1995) is an advantage not shared by many central place foragers such as nesting birds. Our results show the utilization of resources of contrasting quality and quantity, which possibly interacts with the distance of the resource from the central place. Studies often consider foraging decisions as two‐way interactions. For example, energy gain being traded off against some other factor such as predation risk. However, foraging animals require multiple nutrients and may integrate many factors simultaneously in their decision making in order to increase their fitness (Houston & McNamara, 2014). Further study of how these factors interact would give us greater understanding of how animals make foraging decisions. Bee and ant colonies would make good model study systems, as the decisions are distributed among many individuals, allowing for greater amounts of data to be gathered.
